# PW03-023 – Role of S100A4 in inflammatory disorders

**DOI:** 10.1186/1546-0096-11-S1-A249

**Published:** 2013-11-08

**Authors:** M Grigorian, MT Hansen, B Forst, D Sevumian, J Klingelhofer, H Hayrapetyan, T Sarkisian, N Ambartsumian

**Affiliations:** 1 University of Copenhagen, Copenhagen, Denmark; 2 Danish Cancer Society, Copenhagen, Denmark; 3 Center of Medical Genetics and Primary Health Care, Yerevan, Armenia

## Introduction

Tight association of the pro-inflammatory events with the pathogenesis of a majority of serious human diseases (neoplasia, neurodegenetarions, cardiovascular and others) in addition to inflammatory and autoinflammatory diseases is firmly documented. S100A4 is a metastasis-promoting gene whose expression has been found implicated in chronic inflammation. Earlier we have shown an immense upregulation of S100A4 in affected tissues and plasma of patients suffering from rheumatoid arthritis, psoriasis, dermato/polymyositis ^1 - 3^, as well as in pro-inflammarory pathways in tumor metastasis ^4,5^. However, its role in inflammation remains unclear.

## Objectives

Objectives of the work were (1) to further explore the involvement of S100A4 in chronic inflammatory disorders in humans and (2) disclose the molecular mechanisms implicating S100A4 in the pathogenesis of inflammation- associated, particularly in cancer progression (metastasis).

## Methods

We have used several approaches to explore the role of S100A4 in proinflammatory pathways stimulatimg cancer progression. These include in *vitro* (q-RT-PCR, Sandwich ELISA and others) , as well as *in vivo* mouse models for validation of the significance of pro-inflammatory factors in tumor metastasis

## Results

We have obtained data demonstrating a significant increase of S100A4 in plasma of patients with Familial Mediterranean Fever (FMF) likely implicating implication of S100A4 in the pathogenesis of the disease. Correlation of the S100A4 concentration in plasma with a pattern of the genetic point mutations in MEFV gene has been analyzed.

Here we will also present data elucidating a putative link of S100A4 with inflammation on the model of cancer progression. We found that the acute phase response proteins Serum Amyloid A (SAA) 1 and SAA3 are transcriptional targets of S100A4 via TLR4/NF-κB signaling. Data obtained demonstrated SAA proteins stimulate their own transcription, as well as that of S100A8, S100A9, RANTES, G-CSF, MMP2, MMP3, MMP9 and MMP13. They strongly enhanced tumor cell adhesion to fibronectin, and stimulated chemotactic migration. Intravenously-injected S100A4 protein induced expression of SAA1 and SAA3 in an organ-specific manner. In a breast cancer animal model, ectopic expression of SAA1 or SAA3 in tumor cells potently promoted wide spread metastasis formation accompanied by a massive infiltration of immune cells.

## Conclusion

These findings suggest that chronic inflammation mediated by S100A4 and SAA proteins can promote metastasis, and thus that therapeutic targeting of such pro-inflammatory pathways should be effective in combating metastatic disease.

## Disclosure of interest

None declared

